# Effects of the Intragastric Balloon MedSil® on Weight Loss, Fat Tissue, Lipid Metabolism, and Hormones Involved in Energy Balance

**DOI:** 10.1007/s11695-014-1191-4

**Published:** 2014-02-01

**Authors:** Marek Bužga, Machytka Evžen, Klvaňa Pavel, Kupka Tomáš, Zavadilová Vladislava, Zonča Pavel, Zdeněk Švagera

**Affiliations:** 1 Department of Physiology and Pathophysiology, Faculty of Medicine, University of Ostrava, Syllabova 19, Ostrava, 703 00 Czech Republic; 2 Department of Clinical Studies, Faculty of Medicine, University of Ostrava, Syllabova 19, Ostrava, 703 00 Czech Republic; 3 Department of Surgical Studies, Faculty of Medicine, University of Ostrava, Syllabova 19, Ostrava, 703 00 Czech Republic; 4 Department of Biomedical Sciences, Faculty of Medicine, University of Ostrava, Syllabova 19, Ostrava, 703 00 Czech Republic

**Keywords:** Intragastric balloon, Leptin, Ghrelin, FGF21, Lipid metabolism, Fat tissue, Weight loss

## Abstract

**Background:**

The prevalence of obesity continues to increase worldwide. Because obesity is associated with a number health-related problems as well as a shortened life span, treating obesity is an important clinical concern. Although various treatments are currently available, many are not efficacious in the long term. Therefore, additional medical treatment options for morbidly obese individuals must be explored. In this study, we examined the effects of the intragastric balloon MedSil® on anthropometric measures and hormones associated with lipid and energy metabolism.

**Methods:**

Twenty-two obese patients underwent insertion of the intragastric balloon MedSil® following a clinical exam, body composition scan, and collection of blood samples. Six months following implantation of the balloon, additional anthropometric and serological measures were taken.

**Results:**

Six months following insertion of the MedSil® balloon, we observed significant decreases in body weight, body mass index, and fat mass. Compared with baseline levels, ghrelin serum levels were increased significantly, while leptin, FGF21, and glycated hemoglobin levels significantly decreased, 6 months after balloon insertion.

**Conclusions:**

The MedSil® intragastric balloon is a safe and effective treatment for morbid obesity, with positive effects on anthropometric measures and lipid metabolism.

## Introduction

The rapidly increasing prevalence of obesity observed in the recent decades may be a cause of major public health problems of a pandemic nature [1]. According to forecasts, there will be 2.3 billion overweight adults and more than 700 million people suffering from obesity worldwide by 2015. The scale of the problem is confirmed by the fact that morbid obesity body mass index (BMI) > 40 kg/m^2^ shortens life span, on average, by 20 years, making the consequences of obesity more severe than those of tobacco smoking or alcohol consumption [2].

The treatment of obese patients is a demanding and long-term undertaking, in which there are no shortcuts or quick fixes. Data from the literature clearly show that no weight loss regiment following pharmacotherapy or diet therapy remains effective in the long run [3]. In patients with morbid obesity (BMI = 40 kg/m^2^), conservative treatment appears ineffective [4]. Today, bariatric surgical treatment at high obesity levels is the most effective procedure with the best outcomes from a long-term perspective. Obese subjects who do not qualify for, or do not give consent to, bariatric surgical procedures constitute a therapeutic problem. An endoscopic method for the treatment of obesity, intragastric balloon, can be an option for this group of patients [5].

Several hormones are involved in the regulation of the energy balance of the body. An excessive accumulation of adipocytes that constitutes a metabolically active tissue, producing biologically active cytokines, plays an important role in lipid and carbohydrate metabolism. Therefore, we evaluated the efficacy of obesity treatment using the intragastric balloon MedSil®. Effect on body composition, serum levels of ghrelin, leptin, adiponectin, angiopoietin-like protein 3 (ANGPTL-3), angiopoietin-like protein 4 (ANGPTL-4), fibroblast growth factor 19 (FGF19), and fibroblast growth factor 21 (FGF21) were examined, as well as the impact of this method on lipid metabolism.

## Methods

### Patients

A group of 22 patients underwent endoscopic treatment by intragastric balloon at the Endoscopy Center of Internal Clinic, University Hospital of Ostrava, between July and December 2012. The patients’ mean age was 45.4 years (range 36.1–61.2); the group comprised eight male and 14 female subjects. Patients were selected by the gastroenterologist in cooperation with the nutrition team. Inclusion criteria were BMI > 30 kg/m^2^ and need for preoperative weight loss. Patients with the following comorbidities at the time of Medsil® placement were excluded: acute gastritis, history of stomach surgery, gastric and duodenal ulcers, hypertension (blood pressure >140/90 mmHg), fasting glycemia (>7.0 mmol/L), respiratory disorders (sleep apnea and/or tachypnea after little physical activity), and hypolipidemic and antidiabetic treatment. The study was approved by the ethical committee at the Faculty of Medicine, University of Ostrava and University Hospital Ostrava, Czech Republic, in accordance with the ethical standards of the Helsinki Declaration of 1975, as revised in 2000.

### Endoscopy

**Fig. 1:**
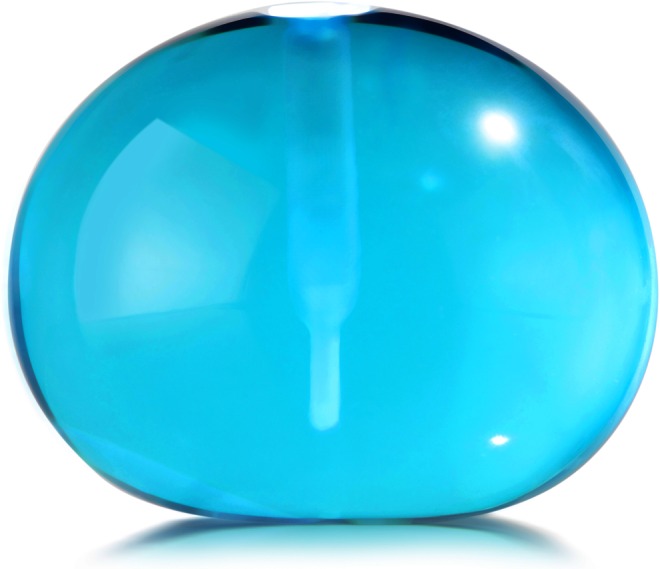
The intragastric balloon MedSil® used in endoscopic treatment for morbidly obese patients

**Fig. 2:**
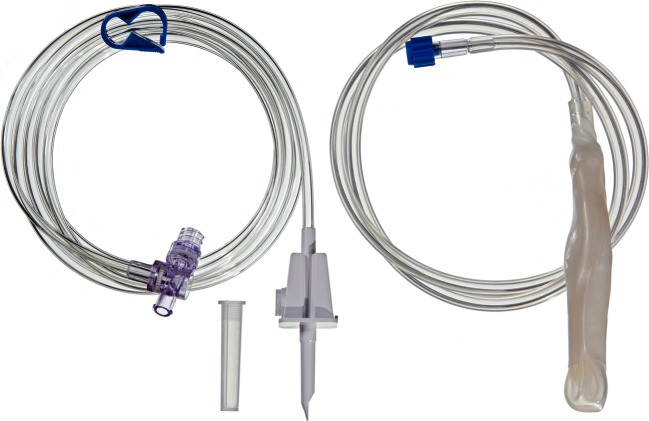
A system of tubes for saline solution in endoscopic treatment of the intragastric balloon MedSil®

For endoscopic treatment, we used the intragastric balloon MedSil® (Figs. 1 and 2). The MedSil® balloon is a saline-filled balloon with a maximal volume of 700 mL. The technical implantation of the MedSil® balloon is almost the same as similar balloons, such as BIB® and Silimed®. The only peculiarity of the placement procedure for the MedSil® intragastric balloon is the recommendation of the manufacturer to treat not only the outer surface of the balloon with lubricant before implantation, but also to introduce lubricant under the sheath. The endoscopist should roll down the distal part of the sheath approximately 1 cm, apply 1 mL of lubricant on the inner surface of the sheath, spread the lubricant with their finger so that the inner part of the sheath is covered with a smooth layer of lubricant, and roll up the distal part of the sheath. This provides easy detachment of the balloon from the filling tube.

Balloons were implanted under sedation with midazolam (5–10 mg) in the outpatient setting using standard techniques as with any other balloon. First, diagnostic upper endoscopy was conducted to exclude any patient with contraindications. Then, patients swallowed the empty balloon. Under endoscopic vision, the balloon was filled with 500 mL of saline. After implantation, the patient stayed in the recovery room for 2 h for observation. Following the procedure, patients took antiemetics, such as metoclopramide and thiethylperazine, at home for a few days.

### Measurements

At the screening visit, subjects had a routine clinical exam, and information concerning medical history and anthropometrics was collected. Height and weight were measured with subjects wearing light clothing and no shoes. A week before the planned intervention, body composition was assessed in all the probands by the dual energy X-ray absorptiometry (DXA) method (Discovery A; Hologic, Waltham, MA, USA). At the same time, venipuncture was performed (morning hours, fasting). Blood samples were processed for subsequent analysis 20 min after venipuncture. Serum concentrations of the following substances were assayed: glucose, ]triacylglycerols, total cholesterol, and high-density lipoprotein and low-density lipoprotein cholesterol (AU 5420, Beckman Coulter, Inc., Brea, CA, USA). To reduce analytical variation, hormones and cytokines of fat tissue from all patients were analyzed in the same run. Blood samples were kept at -80 °C until the time of analysis. Serum levels of leptin and adiponectin were analyzed by enzyme-linked immunosorbent assay (ELISA) using sandwich sets (Biovendor-Laboratorni Medicina, Brno, Czech Republic). Serum concentrations of the following substances were assayed with commercially available ELISA kits: ANGPTL-3, ANGPTL-4, and FGF19 (Biovendor-Laboratorni Medicina). FGF21 was determined by a multiplex assay (Biovendor-Laboratorni Medicina) using barcoded magnetic beads and performed on a Biocode-100A (Applied BioCode Inc., Santa Fe Springs, CA, USA).

Following the results of these assays, the intragastric balloon was endoscopically placed in the stomach. Three and 6 months after the endoscopy, individuals were invited for a follow-up including a clinical exam, DXA, and collection of blood samples.

### Statistical Analysis

All the statistical tests were evaluated at the significance level of 5 %. A *p* value of <0.05 was considered statistically significant. Because of a non-Gaussian distribution of most of the data, the logarithm and square root were used for transforming data to achieve normality with constant variance across observations. The Shapiro-Wilk test was used to test normality of the data. Experiments were conducted as repeated measurements in time over different persons. As such, autocorrelation of the measurements among the same patient was assumed. Therefore, we used linear mixed models (lme, part of nlme package) where each patient represented a random effect [6]; otherwise, incorrect degrees of freedom and error terms would be estimated. The effects of gender and intragastric balloon in the full model were tested using the *F* test. The *t*-statistic was used to test if each particular coefficient of explanatory variables was equal to zero. These analyses were performed using *R* [7].

## Results

During the 48–72 h following endoscopy, several patients experienced mild abdominal pain, nausea, and vomiting. These symptoms were easily controlled by medical therapy in all cases. The severity and time of nausea and vomiting after implantation were similar to those with other types of balloons [8]. After 6 months, the balloon was endoscopically removed from the stomach in 22 patients. No serious complications (mortality, ulceration, bleeding from the alimentary tract, perforation, intestinal obstruction, electrolyte disorders, and deflation of the balloon) were observed during the course of treatment with the MedSil® balloon.

The group of 22 patients had a mean BMI of 43.3 kg/m^2^ with a mean weight of 128.5 kg (from 85 to 190 kg). Using DXA, the average percentage of fat at study onset was 43.4 % (from 32 to 58 %). The balloon placement caused a significant body weight reduction within a period of 6 months. At the end of the treatment, the BMI decreased to 37.8 kg/m^2^ (SD 9.4; *p* < 0.001), with a mean weight of 110.1 kg (SD 27.8; *p* < 0.001). The mean weight loss was 18.4 kg (SD 8.2; *p* < 0.032). The excess weight loss (EWL) was 19.3 % (20.9 % in women and 16.3 % in men) 6 months following endoscopy. The excess BMI loss (EBL) was 26.3 % (30.4 % in women and 18.7 % in men). Excess weight, BMI loss, and fat and lean body mass data are summarized in Table 1. The application of the intragastric balloon caused a significant decrease in glycated hemoglobin (*p* < 0.036), but not in fasting glucose. The data of excess serum parameters of lipids and glucose are summarized in Table 2.

**Table 1:** Overview of weight and body composition parameters in all patients

*N* = 22	Baseline	3 months	6 months	*p* value
Weight (kg) (min–max)	128.5 ± 33.5 (85–190)	120.2 ± 32.8 (79–181)	110.1 ± 27.8 (72–178)	<0.001
Weight loss (kg)		8.3 (1–23)	18.4 (1–33)	<0.032
Fat (kg) (min–max)	56.9 ± 21.9 (32–105)	51.5 ± 20.9 (30–98)	45.2 ± 18.5 (29–96)	<0.001
Fat (%) (min–max)	43.4 ± 7.4 (32–58)	41.4 ± 7.4 (29–55)	39.4 ± 7.4 (28–55)	<0.001
LBM (kg) (min–max)	71.9 ± 14.3 (49–98)	69.4 ± 13.3 (48–94)	66.6 ± 14.9 (45–96)	<0.0001
BMI (kg/m^2^) (min–max)	43.3 ± 10.6 (28.9–65.4)	40.4 ± 10.4 (26.8–62.7)	37.8 ± 9.4 (25.1–60.2)	<0.001
EBL (%) (min–max)		20.0 ± 14.7 (1.1–71.8)	26.3 ± 24.3 (2.8–98.4)	
EWL (%) (min–max)		10.8 ± 7.3 (0.78–33.6)	19.3 ± 12.7 (2.6–50.3)	

Data are expressed as mean ± SD. *p* values refer to significantly different values between baseline and 6 months following surgery (*F* test)

*LBM* lean body mass, *EBL* excess BMI loss, *EWL* excess weight loss

**Table 2:** Overview of serum parameters of lipid and glucose metabolism

*N* = 22	Baseline	3 months	6 months	*p* value
Fasting glucose (mmol/L)	5.7 ± 0.6 (4.9–7.0)	5.5 ± 0.5 (4.7–6.8)	5.5 ± 0.7 (4.6–7.8)	0.075
HbA1c (mmol/mol)	44 ± 10 (35–71)	39 ± 5.0 (33–55)	39 ± 4.0 (31–50)	0.008
TC (mmol/L)	5.7 ± 0.9 (4.6–8.9)	5.4 ± 1.1 (3.4–8.7)	5.6 ± 1.2 (3.8–8.2)	0.481
TG (mmol/L)	2.0 ± 1.0 (0.9–4.9)	1.8 ± 0.9 (0.7–4.4)	1.9 ± 1.4 (0.8–5.7)	0.267
HDL (mmol/L)	1.2 ± 0.3 (0.8–1.8)	1.2 ± 0.3 (0.8–1.9)	1.2 ± 0.2 (0.9–1.6)	0.870
LDL (mmol/L)	4.1 ± 0.8 (3.1–6.4)	3.8 ± 0.9 (2.3–6.4)	3.9 ± 0.9 (2.3–5.9)	0.106

Data are expressed as mean ± SD. *p* values refer to significantly different values between baseline and 6 months following surgery (*F* test)

Ghrelin was found to increase significantly 3 and 6 months after the balloon insertion. Among the plasma adipokines, leptin significantly decreased from 30.4 to 14.9 μg/L (SD 15; *p* < 0.001). At baseline, the levels of adiponectin did not differ significantly. An interesting response was shown in plasma levels of FGF21. There was a rise in plasma levels 3 months after insertion of the balloon, with a consequent significant decrease after 6 months. The changes in ANGPTL-3 and ANGPTL-4 were not statistically significant 6 months following endoscopy. The data of serum parameters of hormonal changes are summarized in Table 3.

**Table 3:** Overview of cytokines and hormones of energy regulation

*N* = 22	Baseline	3 months	6 months	*p* value
Ghrelin (μg/L)	240.5 ± 101.5 (21.74–380.1)	378.1 ± 155.8 (171.0–658.8)	335.8 ± 149.2 (145.0–703.2)	<0.002
Leptin (μg/L)	30.4 ± 17.2 (6.9–54.5)	18.2 ± 15.8 (5.7–50.7)	14.9 ± 15.5 (4.7–50.3)	<0.001
Adiponectin (mg/L)	17.9 ± 9.0 (7.0–33.5)	16.9 ± 9.1 (7.0–42.8)	20.5 ± 10.2 (5.8–36.2)	0.285
FGF19 (ng/L)	148.7 ± 132.3 (42.3–621.0)	188.4 ± 78.9 (60.1–344.0)	173.6 ± 73.4 (86.8–340.6)	0.111
FGF21 (ng/L)	68.2 ± 48.1 (6.0–151.9)	68.9 ± 62.8 (3.8–231.7)	49.9 ± 56.8 (3.0–204.0)	<0.002
ANGPTL-3 (μg/L)	295.9 ± 75.8 (174.0–470.0)	286.3 ± 82.6 (132.0–417.0)	346.1 ± 99.1 (122.0–498.0)	0.163
ANGPTL-4 (μg/L)	82.4 ± 20.7 (55.7–131.0)	78.1 ± 19.1 (51.1–108.3)	87.7 ± 29.6 (34.7–141.2)	0.578

Data are expressed as mean ± SD. *p* values refer to significantly different values between baseline and 6 months following surgery (*F* test)

## Discussion

The intragastric balloon has been shown to be a safe and effective procedure for temporary weight reduction, with low mortality and morbidity [8]. The use of intragastric devices to support weight reduction is not novel [9]. Over the years, several intragastric balloons filled with air and fluid have been developed, which are less invasive than surgical treatment for morbid obesity. For several years, only the Bioenterics Intragastric Balloon (BIB) was used and approved according to physician feedback, and only recently have other similar devices been commercialized [10]. Intragastric balloons have played an essential role in the preoperative treatment of morbidly obese patients who are scheduled to undergo bariatric or other elective surgery by minimizing mortality and morbidity risks [11]. In our study, we used an intragastric balloon similar to the BIB. In terms of clinical complications, the balloons showed a very good tolerance in patients enrolled in the study.

We demonstrated a beneficial effect of MedSil® balloon on body composition. The placement of the intragastric balloon for 6 months resulted in a statistically significant reduction in body weight. The mean losses of weight and BMI were 18.4 kg and 5.5 kg/m^2^, respectively. Our results are comparable to previous reports in which the weight loss was 14.7–17.8 kg and BMI loss was 5.7–6.7 kg/m^2^ [12–14]. Similar results were obtained by others [5, 15–18], using various types of intragastric balloons. The mean weight loss fluctuated from 9.7 to17.8 kg in these studies 6 months following balloon insertion [5, 12–18]. The fat mass decreased on average by 11.7 kg. Loss of fat-free mass was about 5.3 kg during the 6-month period. The loss of body fat is the most important objective of obesity treatment. However, the current decline of fat-free mass is often observed. Maintenance of fat-free mass is of particular importance in obesity treatment to minimize the reduction in energy expenditure seen after weight loss. This may be a result of the negative energy balance, lower body weight, and less stimulation for muscle growth in the lower limbs [19–21].

We demonstrated a positive effect of the intragastric balloon MedSil® on glucose tolerance. The observed improvement of glycated hemoglobin levels was statistically significant. However, no significant changes were reported in fasting glucose levels. Decreases in glycated hemoglobin in patients with BIB were reported in several studies. Sekino describes a decrease in glycated hemoglobin; however, this decrease was not statistically significant [22]. In a 6-month study with BIB, Konopko-Zubrycka documented a significant decrease in fasting glucose and insulin response. Similar results were seen by Mathus-Vliegen in their randomized study [5, 23].

There are few available reports on the effect of gastric balloons on hormonal regulation in patients with morbid obesity [5, 22, 24]. Body weight is regulated by a complex system, including both peripheral and central factors. Two of the hormones that seem to play an important role in the regulation of food intake and body weight are leptin and ghrelin [25, 26]. Ghrelin, a 28-amino acid peptide secreted mainly by the stomach [27], acts as an orexigenic molecule. Ghrelin stimulates both energy gain and the secretion of growth hormone (GH) and insulin leading to weight gain and attainment of a positive energetic balance in the long term [28]. In addition, ghrelin levels seem to be influenced by age, gender, BMI, growth hormone, glucose, and insulin [29]. The effect of leptin, a 167-amino acid protein produced by adipocytes, in the hypothalamus is opposite to that of ghrelin; in other words, leptin acts as an anorexigenic molecule [30]. Leptin is proportionally released to the amount of fat stored in the white adipose tissue and acts in hypothalamic suppression of food intake and increase in energy expenditure [31]. In the present study, the levels of ghrelin increased significantly 3 months following the insertion of the intragastric balloon. Subsequently, the levels of ghrelin decreased but were still above the values measured at baseline. These findings are in concordance with the data obtained by Konopko-Zubrycka et al., [5] who found increases in plasma ghrelin 1 and 6 months after the insertion of an intragastric balloon. Mion et al. reported similar effects on the levels of ghrelin in air-filled intragastric balloons in non-morbidly obese patients [32]. Leptin showed a significant decrease 6 months after the insertion of the balloon which is probably related to the decrease in the amount of adipose tissue after MedSil® balloon application. The decline in plasma leptin levels was similarly reported in three studies with the introduction of the BIB in morbidly obese patients [5, 22, 29].

Six months following the intragastric balloon insertion, we found significant decreases in FGF21 levels, but not in FGF19 levels. FGF21 was considered a metabolic hormone regulated by nutritional status, with beneficial effects on glucose homeostasis and lipid metabolism in animal models [33]. In humans, increased FGF21 levels are associated with obesity in both children [34] and adults [35, 36], indicating a connection between FGF21 and body fat mass. Studies that have analyzed the response of FGF21 to weight loss in humans have shown controversial findings. Mai et al. [37] showed that moderate weight loss (∼5 kg) induced changes in FGF21 levels in 30 obese subjects following a hypocaloric diet and physical activity regimen for 6 months. The same results were published in 23 non-diabetic, morbidly obese subjects 1 year after laparoscopic Roux-en-Y gastric bypass and laparoscopic sleeve gastrectomy [38]. However, a recently published work describes significant decreases in levels of FGF21 in 17 obese females undergoing laparoscopic sleeve gastrectomy [39], which is in accordance with our results.

The current study has its limitations. The patient group was relatively small and follow-up in the evaluation of effectiveness after balloon removal was limited to 6 months. Data characterizing the evolution of comorbidities in these subjects were also limited. An interesting question for further study is the extent of weight loss achieved after balloon removal. Dastis et al. report maintenance of >10 % weight loss in 25 % of patients for up to 2.5 years after BIB balloon removal [40]. Given the similarities between the BIB and MedSil® balloons, we assume that our patients would achieve similar results. Our study is ongoing and we plan to prospectively monitor these patients for 18 months after balloon removal.

## Conclusion

The MedSil® intragastric balloon system is a safe and effective method, with minimal clinical complications, in the treatment of morbidly obese patients. The method of using the intragastric balloon is perceived as a restrictive surgery, especially suitable for patients before bariatric surgical treatment. Our experience shows that the method is not purely restrictive. Intragastric balloon has a positive effect on glucose homeostasis and the molecules regulating lipid and energy metabolism.
